# PGLYRP2 as a novel biomarker for the activity and lipid metabolism of systemic lupus erythematosus

**DOI:** 10.1186/s12944-021-01515-8

**Published:** 2021-08-30

**Authors:** Hui Li, Defang Meng, Jieting Jia, Hua Wei

**Affiliations:** Northern Jiangsu People’s Hospital, Affiliated Hospital to Yangzhou University, Yangzhou, Jiangsu 225002 People’s Republic of China

**Keywords:** Systemic lupus erythematosus, SLE disease activity index, PGLYRP2, Lipid profile

## Abstract

**Background:**

To assess the value of peptidoglycan recognition protein 2 (PGLYRP2) in assessing the disease activity and lipid metabolism in patients with systemic lupus erythematosus (SLE).

**Methods:**

SLE patients with stable disease (*n* = 15), active lupus nephritis (LN) (*n* = 15) and neuropsychiatric systemic lupus erythematosus (NP-SLE) (*n* = 15) admitted to Northern Jiangsu People’s Hospital (Jiangsu, China) in 2019–2020 were recruited. In addition, volunteers with matched age and sex (*n* = 15) were enrolled as controls. The level of PGLYRP2 in the serum and its expression in peripheral blood mononuclear cells (PBMCs) were measured. The link between PGLYRP2 level and clinical parameters (including lipid profile) was described.

**Results:**

Serum PGLYRP2 level in SLE cases exceeded that in healthy volunteers (3938.56 ± 576.07 pg/mL), and significantly higher in active LN (5152.93 ± 446.13 pg/mL) and NP-SLE patients (5141.52 ± 579.61 pg/mL). As shown by quantitative real-time PCR results, the expression of PGLYRP2 in PBMCs of SLE patients with active LN and NP-SLE surpassed that in healthy volunteers (*P* < 0.01). Receiver operating characteristic (ROC) curves demonstrated that PGLYRP2 was capable of distinguishing stable SLE from active LN (AUC = 0.841, 95%CI = 0.722–0.960, *P* = 0.000). PGLYRP2 level positively correlated with SLEDAI of SLE patients (r = 0.5783, *P* < 0.01). Moreover, its level varied with serological and renal function parameters (complement 3, complement 4, estimated glomerular filtration rate and 24-h urine protein) and immunoglobulin A (IgA) of SLE. A potential correlation between PGLYRP2 level and lipid profile (HLD-c, Apo-A1 and Apo B/A1) was determined in SLE patients. The linear regression analysis indicated SLEDAI as an independent factor of PGLYRP2 level, with a positive correlation in between (*P* < 0.05).

**Conclusions:**

Serum PGLYRP2 level significantly increases in SLE patients, and is positively correlated to SLEDAI. Moreover, serum PGLYRP2 level is correlated with renal damage parameters and the abnormal lipid profile of SLE. PGLYRP2 could be used to predict SLE activity, dyslipidemia and cardiovascular disease risks in SLE patients.

## Introduction

Systemic lupus erythematosus (SLE) features lupus nephritis (LN) [1]. At present, the clinical application of conventional indexes for assessing SLE, like anti-dsDNA antibody, complement 3 (C3), complement 4 (C4), is limited by their unsatisfactory sensitivities and specificities. Novel biomarkers with high efficacy and accuracy are urgently required for clinical assessment of SLE, especially for the disease activity and organ involvement of SLE. Peptidoglycan recognition protein 2 (PGLYRP2) is an innate immune pattern recognition receptor specifically expressed in the liver. Through hydrolyzing the amide bond between the N-acetylmuramic acid and L-Ala, PGLYRP2 separates the cross-linked bacterial peptidoglycans (PGN) and thus digests them into fragments. It is reported that PGLYRP2 is functional in tumor development, immune response in tumor microenvironment, infectious diseases, inflammatory responses and brain development [2–4]. To date, no studies have described the profile of PGLYRP2 in the case of SLE. Therefore, this study aimed to examine serum PGLYRP2 level in SLE cases, and explore its link with disease activity and clinical indexes. PGLYRP2 may be a promising biomarker for predicting the prognosis of SLE.

## Materials and methods

### Subjects

A total of 45 SLE patients (average age, 36.6 ± 13.4 years; range, 18–69 years) were recruited from Northern Jiangsu People’s Hospital from January 1, 2019 to November 1, 2020, including 39 (86.6%) females and 6 (13.4%) males. Inclusion criteria: (1) Age > 18 years; (2) SLE was diagnosed and categorized based on *the 1982 American College of Rheumatology Revised Criteria for Classification of Systemic Lupus Erythematosus* [5]. Exclusion criteria: (1) Combined with other rheumatic immune diseases, tumors, or infection diseases; (2) Pregnant women; (3) Out of a normal BMI range; (4) Taking antihyperlipidemic agents. Recruited SLE patients were averaged into three groups as follows: SLE with stable disease (SLEDAI ≤4, *n* = 15), SLE with active LN (SLEDAI > 5, *n* = 15) and SLE with NP-SLE (SLEDAI > 5, *n* = 15). Using the new criteria of American College of Rheumatology (ACR) for SLE classification, active LN was confirmed with the results of renal biopsy before or at admission, and 24-h urine protein ≥0.5 g; NP-SLE was diagnosed by the presence of at least one of the 19 neuropsychiatric syndromes in SLE and no other explanations for SLE. Diagnosis of complications like hyperlipidemia and hypertension was made according to the relevant guidelines [6, 7]. In addition, 15 healthy volunteers with comparable age and sex were recruited during the same period. This study conformed to the Declaration of Helsinki. Informed consent was obtained before the initiation of this study, and experimental procedures were approved by the Ethics Committee of the Northern Jiangsu People’s Hospital of Jiangsu Province (NO.2021ky155, 2019 to 2020).

### Determination of SLE activity

SLE activity was recorded by Systemic Lupus Erythematosus Disease Activity Index 2000 (SLEDAI) score. Recruited SLE patients were further categorized to low disease activity group (SLEDAI ≤9, *n* = 19), moderate disease activity group (10 < SLEDAI < 14, *n* = 12) and high disease activity group (SLEDAI ≥15, *n* = 14).

### Laboratory test

Morning venous blood and urine were sampled after 8-h fasting, and stored at − 80 °C for urinalysis, and measurement of serum creatinine (SCr), estimated glomerular filtration rate (eGFR), Uric acid, 24-h urine protein, C3, C4, anti-dsDNA antibody, anti-nuclear antibody (ANA), low density lipoprotein cholesterol (LDL-c, calculated using the Friedewald equation), high density lipoprotein cholesterol (HDL-c), apoprotein (apo) A1 (Apo-A1), apoprotein B (Apo-B), immunoglobulin A (IgA), immunoglobulin M (IgM), immunoglobulin G (IgG).

### Isolation of PBMCs

Peripheral blood (2 ml) was collected from each participant and diluted in PBS. An equal volume of Ficoll was slowly added into the Falcon tube alongside the wall, and the mixture was centrifuged at 500×g for 20 min at room temperature. The middle layer was carefully transferred to a new tube, and washed in PBS twice. After centrifugation, the precipitant containing PBMCs was lysed in Trizol reagent (Vazyme Biotech,Nanjing, China) for RNA extraction.

### Real-time PCR

Total RNA was reversely transcribed to cDNAs using the HiScript II Q RT SuperMix (Vazyme Biotech, Nanjing, China). Later, PCR was implemented on the SYBR Green Master Mix and the StepOnePlus Real-Time PCR System (Applied Biosystems, Foster City, CA). Relative level was calculated by the 2^–ΔΔCt^ method, with GAPDH as the internal reference. Gene-specific primers (Genscript Biotech, Nanjing, China) are listed as below. 5′-GCACTTCACCGCGACTGTTA-3′ as the PGLYRP2 forward primer sequence and 5′-TTATTGGAGGTCTGTGGCTGG-3′ as the reverse primer sequence resulted in a PCR product of 103 bp. 5′-TCAGTGGTGGACCTGACCTG-3′ as the GAPDH forward primer sequence and 5′-TGCTGTAGCCAAATTCGTTG-3′ as the reverse primer sequence resulted in a PCR product of 244 bp.

### Elisa

Blood was sampled from each participant and centrifuged at 1000×g for 10 min. The supernatant was moved into a new tube, subpackaged and stored at − 80 °C. Each sample was only recycled and thawed for only one time, to prevent protein degradation. Before measurement of serum level of PGLYRP2, serum samples were diluted 1:10. Serum level of PGLYRP2 was tested with Human PGLYRP2 ELISA kit (Biomatik, EKU06521, Ontario, Canada). The optical density at 450 nm was tested with a microplate reader. Each sample was tested twice in a blind way. Serum PGLYRP2 was expressed with pg/mL.

### Statistical analyses

Clinical data and laboratory test results were recorded in the Excel, and data processing was performed using SPSS 21.0. Statistical significance for group comparison was determined at α = 0.05. The normality of measurement data was determined by the Shapiro-Wilk test. Normally distributed measurement data were expressed as $\overline{x}$ ± s, and those in three or more groups were compared by the F-test, followed by LSD-t test. Otherwise, non-normally distributed measurement data were expressed as P50 (P25, P75), and compared by the Bonferroni correction. Enumeration data were shown as n (%), and analyzed through the Chi-square test. A linear regression model was constructed to tease out the independent risk factors of PGLYRP2 level. Its diagnostic power was illustrated by ROC curves.

## Results

### Serum levels and gene expression levels of PGLYRP2 in PBMC

**Fig. 1:**
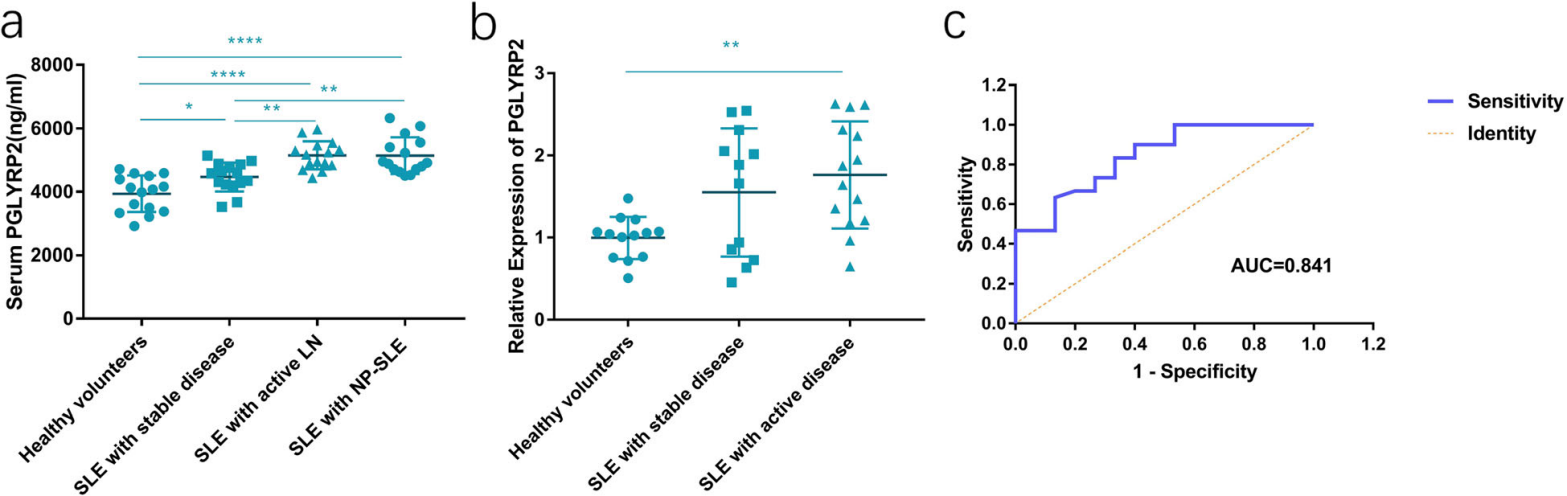
PGLYRP2 level increased in SLE patients. (**A**) Serum level of PGLYRP2 was significantly higher in SLE patients with stable disease (4468.99 ± 457.02 pg/ml), and those with active LN (5152.93 ± 446.13 pg/ml) or NP-SLE (5141.52 ± 579.61 pg/ml), than that healthy volunteers (3938.56 ± 576.07 pg/ml). (**B**) PGLYRP2 level in PBMCs was significantly higher in SLE patients with active LN or NP-SLE than that of healthy volunteers. (**C**) Receiver operating characteristic (ROC) curves showed that serum PGLYRP2 was capable of distinguishing SLE patients with active disease from those with stable disease (AUC = 0.841, 95%CI = 0.722–0.960, *P* = 0.000). *P < 0.05, ** < 0.01, ****P < 0.0001

A total of 45 eligible SLE patients (15 with stable SLE, 15 with active LN and 15 with active NP-SLE) and 15 healthy volunteers (Table 1) were enrolled. Among 45 SLE patients were 39 (86.6%) females and 6 (13.4%) males, with an average age of 36.53 ± 13.54 years. ANOVA's post-hoc test LSD-t test showed that compared with that in healthy volunteers (3938.56 ± 576.07 pg/mL), serum PGLYRP2 was significantly higher in cases with stable SLE (4468.99 ± 457.02 pg/mL), active LN (5152.93 ± 446.13 pg/mL) or NP-SLE (5141.52 ± 579.61 pg/mL) (Fig. 1A). Moreover, serum PGLYRP2 was higher in active LN patients than in stable SLE patients, but showing no significant difference between active LN and NP-SLE cases (*P* > 0.05). Furthermore, it is shown that PGLYRP2 expression level was higher in active LN or NP-SLE patients than in healthy volunteers (*P* < 0.01) (Fig. 1B).

**Table 1:** Baseline information of SLE patients (*n* = 45)

Baseline characteristics	ALL	stable	active	NP-SLE	*P*
n	45	15	15	15	
Age (years)	36.53 ± 13.54	38.00 ± 13.46	32.53 ± 11.60	39.07 ± 15.30	0.375
Female (n)	39	13	13	13	
Median SLEDAI (interquartile)	12.0 (3.0, 17.0)	2.0 (0,3.0)	12.0 (10.0,20.0)	15.0 (12.0,22.0)	< 0.001
Laboratory tests					
Positive ANA (n)	45	15	15	15	–
Positive anti-dsDNA (n)	28	8	9	11	0.516
Hypocomplementaemia (n)	24	6	7	10	0.315
LDL-c (mmol/L)	2.85 ± 1.48	2.32 ± 0.86	3.02 ± 2.21	2.73 ± 1.33	0.478
HDL-c (mmol/L)	1.12 ± 0.43	1.16 ± 0.39	1.37 ± 0.80	1.11 ± 0.43	0.416
Triglycerides (mmol/L)	1.93 ± 1.20	1.53 ± 0.81	2.62 ± 1.70	1.76 ± 1.09	0.056
Apo-A1 (g/L)	0.95 ± 0.31	1.04 ± 0.32	0.89 ± 0.27	0.98 ± 0.33	0.413
Apo-B (g/L)	0.88 ± 0.38	0.69 ± 0.24	0.98 ± 0.43	0.89 ± 0.35	0.078
SCr (μmol/L) (interquartile)	59.0 (45.5104.5)	52.0 (45.0,59.0)	75.0 (50.0,153.0)	47.0 (66.0,107.0)	0.406
eGFR(ml/min∙1.73m^2^) (interquartile)	62.0 (50.0,85.8)	52.0 (45.0,58.0)	76.0 (54.0,120.0)	66.0 (58.3103.3)	0.055
24-h urine protein (mg/L) (interquartile)	294.0 (86.0,1219.0)	170.0 (111.0,831.6)	568.0 (61.0,2126.0)	202.0 (471.0,5036.5)	0.646
C3 (g/L) (interquartile)	0.82 (0.59,1.03)	0.98 (0.86,1.10)	0.62 (0.46,9.00)	0.80 (0.52,1.01)	0.059
C4 (g/L) (interquartile)	0.16 (0.08,0.24)	0.19 (0.12,0.23)	0.10 (0.04,0.43)	0.15 (0.08,0.24)	0.372
Comorbidities					
Hypertension (n)	15	3	7	5	0.301
Diabetes mellitus (n)	2	0	2	0	1.000
Hyperlipidaemia (n)	9	4	4	1	0.235
PAH (n)	8	2	3	3	0.854
Current medications					
Prednisone (n)	43	15	13	15	1.000
Mycophenolate mofetil (n)	15	4	7	4	0.407
Cyclophosphamide (n)	12	5	4	3	0.709
Azathioprine (n)	6	2	3	1	0.549
Hydrochloroquine (n)	44	15	14	15	1.000
Cyclosporin/tacrolimus(n)	8	3	4	1	0.307
ACE inhibitors/ARB (n)	28	8	10	10	0.685

Normally distributed measurement data were shown as $\overline{x}$ ± s, and analyzed by the F-test. Otherwise, they were expressed as P50 (P25, P75), and compared by the Bonferroni correction. Enumeration data were shown as n (%), and analyzed through the Chi-square test

ANA, anti-nuclear antibody; LDL-c, low-density lipoprotein cholesterol; HDL-c, high-density lipoprotein cholesterol; Apo-A, apolipoprotein A; Apo-B, apolipoprotein B; SCr, serum creatinine; eGFR, estimated glomerular filtration rate; PAH, pulmonary arterial hypertension; ACE, angiotensin converting enzyme; ARB, angiotensin receptor blocker

Next, the diagnostic potential of PGLYRP2 in SLE was tested by depicting ROC curves. As shown in Fig. 1C, PGLYRP2 was sensitive and specifical in distinguishing active from stable SLE patients (AUC = 0.841, 95%CI = 0.722–0.960, *P* = 0.000, sensitivity = 63.3%, specificity = 86.7%, Youden index = 0.5).

### Link between serum PGLYRP2 and SLE activity

**Fig. 2:**
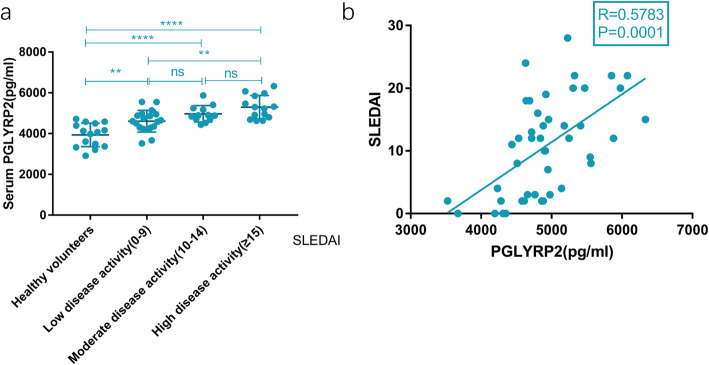
Correlation between serum PGLYRP2 and disease activity of SLE. (**A**) Serum PGLYRP2 was significantly higher in high disease activity group (SLEDAI ⩾15, 5299.94 ± 570.87 pg/ml) than that in low disease activity group (SLEDAI⩽9, 4610.64 ± 533.59 pg/ml). (**B**) Serum level of PGLYRP2 was positively correlated with SLEDAI in SLE patients (r = 0.5783, *P* < 0.01)

Recruited SLE patients were categorized to low disease activity group (SLEDAI ≤9, *n* = 19), moderate disease activity group (10 < SLEDAI < 14, *n* = 12) and high disease activity group (SLEDAI ≥15, *n* = 14). As shown in Fig. 2A, serum PGLYRP2 in high disease activity group (5299.94 ± 570.87 pg/mL) was higher than that in low disease activity group (4610.64 ± 533.59 pg/mL) (*P* < 0.01). However, no significant difference in serum PGLYRP2 was witnessed between moderate disease activity group (4970.85 ± 402.61 pg/mL) and high disease activity group (5299.94 ± 570.87 pg/mL) (*P* > 0.05).

In addition, a positive relationship was identified between serum PGLYRP2 and SLEDAI in SLE patients (r = 0.5783, *P* < 0.01, *n* = 45) (Fig. 2B).

### Link between serum PGLYRP2 and serological and renal function parameters in SLE patients

**Fig. 3:**
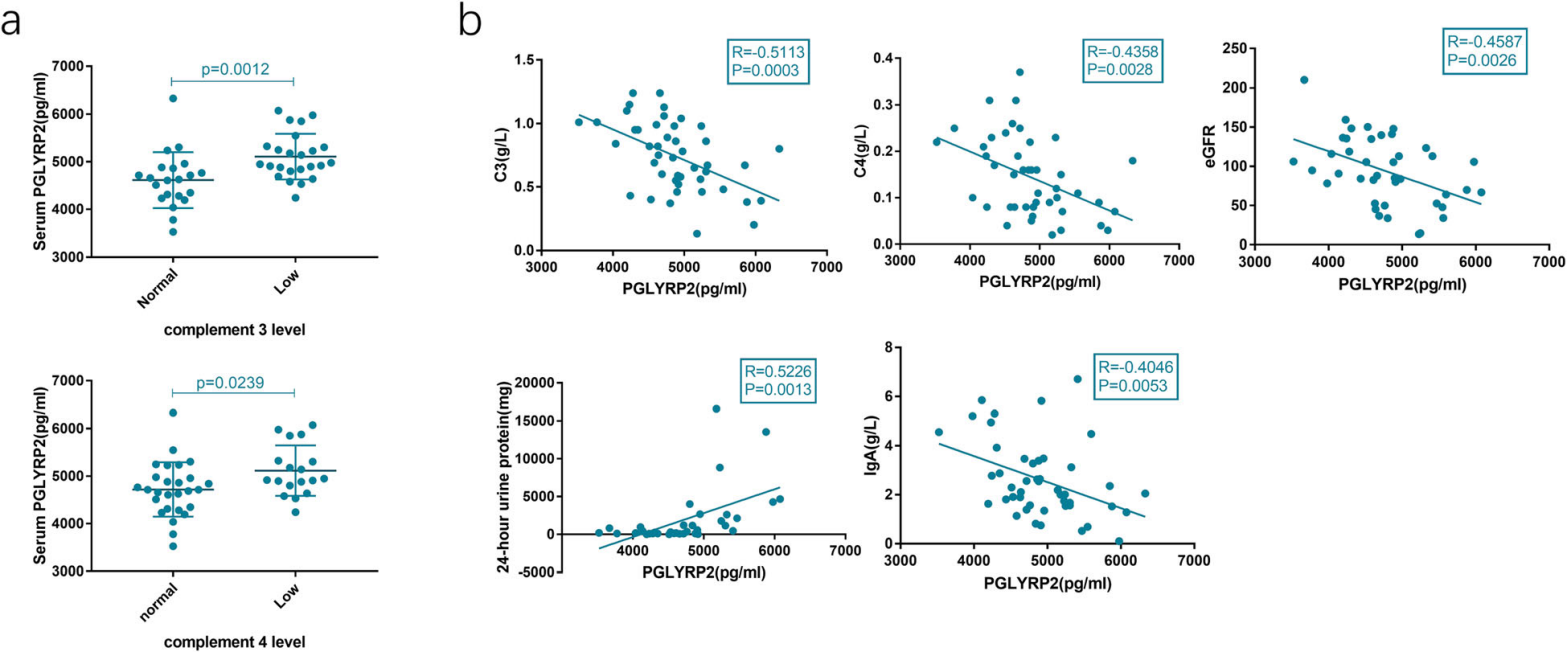
Correlation between serum level of PGLYRP2 and serological and renal function indexes in SLE patients. (**A**) Serum PGLYRP2 was significantly higher in SLE patients with low C3 level (5109.06 ± 479.70 pg/ml) than in those with normal C3 levels (4613.82 ± 588.89 pg/ml) (left side). Serum PGLYRP2 was significantly higher in SLE patients with low C4 level (5116.02 ± 531.27 pg/ml) than in those with normal C4 levels (4719.24 ± 571.00 pg/ml) (right side). (**B**) Serum PGLYRP2 was negatively correlated with C3 level (r = 0.5113, *P* < 0.01), C4 level (r = 0.4358, *P* < 0.01), eGFR (r = 0.4587, *P* < 0.01) and lgA (r = 0.4046, *P* < 0.01), but positively correlated with 24-h urine protein level (r = 0.5226, *P* < 0.01) in SLE patients

The potential correlation of serum PGLYRP2 with serological and renal function parameters of SLE patients was analyzed. Serum C3 and C4 levels were categorized into low or normal. Reduced serum C3 or C4 level was defined as a low complement level. It is shown that serum PGLYRP2 was evidently higher in SLE patients with low-level C3 or C4 than in those with normal ranges (Fig. 3A). However, a significant difference in serum PGLYRP2 was not detected between SLE patients with normal and abnormal ranges of ANA and anti-dsDNA antibodies.

Serum PGLYRP2 was negatively correlated with C3 level (r = 0.5113, *P* < 0.01), C4 level (r = 0.4358, *P* < 0.01), and eGFR (r = 0.4587, *P* < 0.01); positively correlated with 24-h urine protein level (r = 0.5226, *P* < 0.01); negatively correlated with IgA (r = 0.4046, *P* < 0.01) (Fig. 3B) in SLE patients.

### Link between serum level of PGLYRP2 and lipid profile in SLE patients

**Fig. 4:**
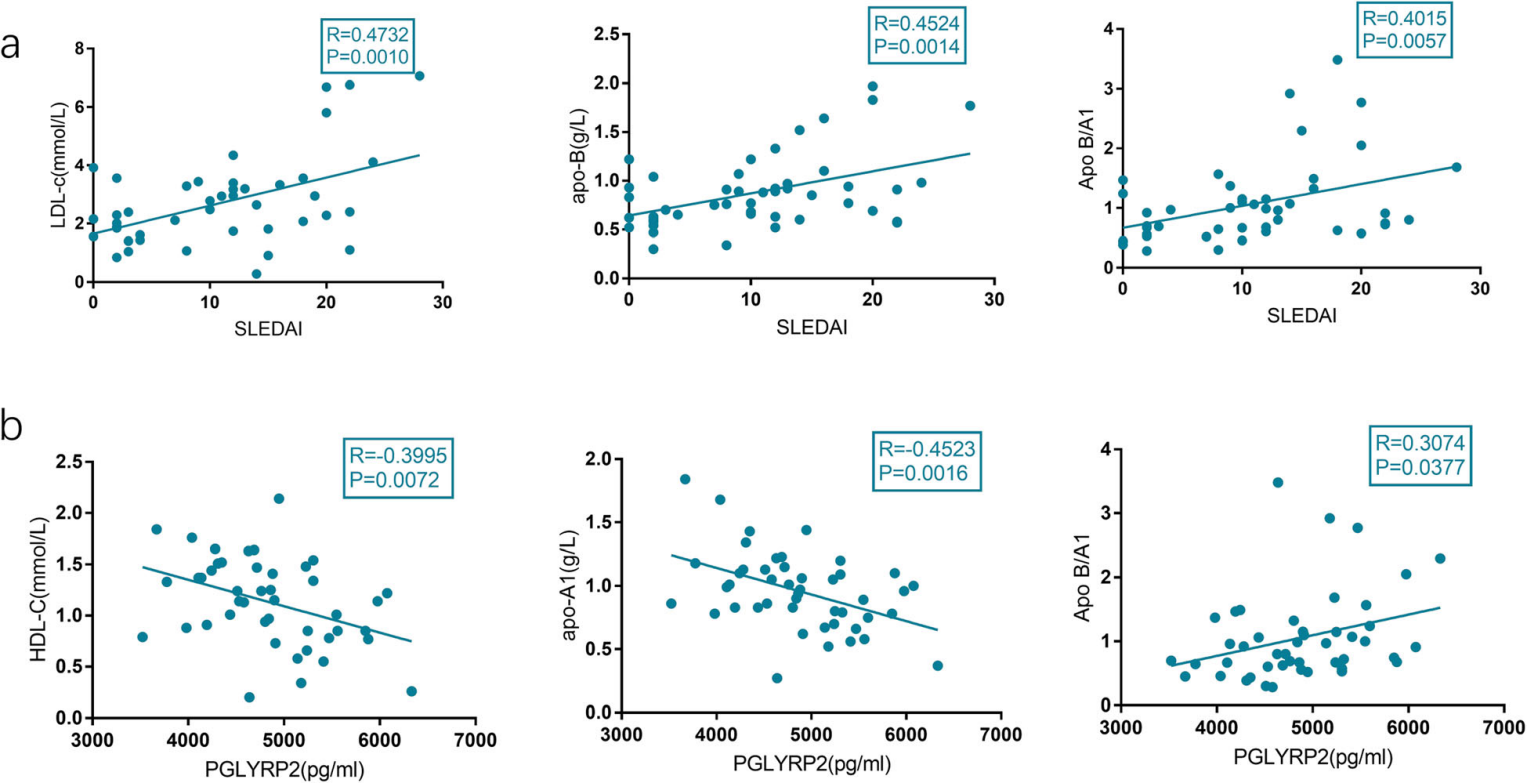
Correlation between serum PGLYRP2 and lipid metabolism indexes in SLE patients. (**A**) SLEDAI was positively correlated with LDL-c (r = 0.47, *P* < 0.01), Apo-B (r = 0.4524, *P* < 0.01), and Apo B/A1(r = 0.4015, *P* < 0.01) in SLE patients. (**B**) Serum PGLYRP2 was negatively correlated with HDL-c (r = 0.3746, *P* < 0.05) and Apo-A1 (r = 0.4523, *P* < 0.01), but positively correlated with Apo-B/A1 (r = 0.3.74, *P* < 0.05) in SLE patients

It is shown that SLEDAI was positively correlated with LDL-c (r = 0.47, *P* < 0.01), Apo-B (r = 0.4524, *P* < 0.01), and Apo B/A1(r = 0.4015, *P* < 0.01) in SLE patients (Fig. 4A). Moreover, serum PGLYRP2 was negatively correlated with HDL-c (r = 0.3746, *P* < 0.05) and Apo-A1 (r = 0.4523, *P* < 0.01), but positively correlated with Apo-B/A1 (r = 0.3.74, *P* < 0.05) in SLE patients (Fig. 4B). PGLYRP2 was linked to the abnormal lipid metabolism in SLE.

The results did not identify a correlation between PGLYRP2 and liver function parameters, like alanine aminotransferase (ALT), aspartate transaminase (AST), gamma-glutamyl transferase (GGT) and bilirubin in SLE patients (data not shown).

### SLEDAI as the independent risk factor of PGLYRP level

To further analyze the potential influences of SLEDAI, C3, C4, eGFR, 24-h urine protein, IgA, HDL-c, apo-A1 and apo-B/A1 on PGLYRP2 level, multiple factor analysis was performed (Table 2). As shown by the linear regression analysis, SLEDAI was the independent risk factor influencing PGLYRP2 level, with a positive correlation in between (*P* < 0.05).

**Table 2:** Independent risk factors influencing PGLYRP2 level

Subject	β	SE	β’	t	P	β (95%CI)	
						Lower	Upper
Constant	4872.328	534.883		9.109	0.000	3768.383	5976.273
SLEDAI	57.199	18.537	0.716	3.086	0.005	18.940	95.459
C3	−1.224	44.267	−0.010	−0.028	0.978	−92.585	90.138
C4	−290.858	1318.002	− 0.073	− 0.221	0.827	− 3011.081	2429.365
GFR	−1.962	3.365	−0.113	−0.583	0.565	−8.908	4.983
24 h urine protein	0.026	0.034	0.124	0.775	0.446	−0.043	0.096
IgA	−45.934	69.554	−0.117	−0.660	0.515	− 189.487	97.618
HDLC	−69.796	194.271	−0.076	−0.359	0.723	− 470.750	331.159
APOA1	−109.037	396.500	−0.061	−0.275	0.786	− 927.372	709.298
apo-B/A1	−41.092	181.885	−0.054	−0.226	0.823	−416.484	334.300

β, beta coefficient; SE, standard error; β’, standardized beta coefficient; β (95%CI), 95% confidence interval of beta coefficient

## Discussion

So far, the pathogenesis of SLE has not been clearly clarified. SLE features overactivation of inflammatory response, production of autoantibodies and activation of complements. Biomarkers that can effectively predict the involvement of kidneys in the early stage of SLE are very useful for design strategies for improving the prognosis of LN. Previous studies have reported that Toll like receptor 9 (Toll-R9), Vascular endothelial growth factor (VEGF), von Willebrand factor (VWF) and A Proliferation-inducing Ligand (APRIL) are novel candidate biomarkers for predicting LN [8–12].

The present study for the first time demonstrated that serum level of PGLYRP2 significantly increased in SLE patients, making it a good predictor for the disease activity of SLE. Moreover, serum level of PGLYRP2 was significantly correlated to SLEDAI, C3 and C4 in SLE patients, although it was not correlated to ANA and anti-dsDNA antibodies. The present study consistently revealed that serum level of PGLYRP2 was significantly correlated to renal function parameters like eGFR and 24-h urine protein in SLE patients, suggesting that the increased serum PGLYRP2 could be a predictor for renal dysfunction and a risk factor for the poor prognosis of SLE.

PGLYRP2 is mainly distributed in the liver, which is released to the blood and responsible for regulating inflammatory response. In human beings, PGLYRP2 is differentially expressed in the liver and skin. It is highly abundant in the liver, but barely expressed in the skin of healthy humans [13]. PGLYRP2 expression is not specific in peripheral blood cells, because it is also expressed in monocytes and T cells. Previous evidence has shown that PGLYRP2 is overexpressed in hepatocellular carcinoma (HCC) cells and can significantly strengthen the anti-tumor immune response in mice. Hence, tumor-derived PGLYRP2 is considered as a novel biomarker for HCC, and its potential in detecting cancer immunity and designing immunotherapies is highlighted [14]. In addition, PGLYRPs can regulate the sensitivity to inflammatory diseases. For example, PGLYRP2 protects against skin inflammation of psoriasis in mice by limiting the overactivation of Th17 cells and accelerating the accumulation of regulatory T cells at the inflammatory site [15]. PGLYRP2 is closely linked to the innate immunity, and regulates chronic inflammation by inhibiting IFN-γ [16]. At present, relevant studies about the role of PGLYRP2 in autoimmune diseases are scant. PGLYRP2 is indispensable in the induction of cytokines, chemokines and receptors in an arthritis model [17]. It is reported that PGLYRP2 can mediate the brain development and behaviors [4]. However, no significant difference in serum PGLYRP2 level was witnessed between active LN and NP-SLE patients.

It is generally believed that cardiac metabolic risk has a close link with the activity of SLE. SLE cases are more prone to cardiovascular events, which is possibly attributed to endothelial damage caused by SLE-induced inflammation. Autoantibodies and circulating immune complexes are involved in the pathogenesis of atherosclerosis by damaging endothelial cells. Increased levels of disintegrins and metalloproteases with a thrombospondin type 1 motif, member 13 (ADAMTS-13), and VWF are causative for cardiovascular events in SLE patients, as well as venous thrombosis. An increase in VWF concentration in high-risk population can predict cardiovascular (CV) outcomes [18–20]. In addition, dyslipidemia is of great significance in the atherosclerosis of SLE patients [21, 22]. The profiles of HDL-c, LDL-c, TG, apolipoproteins and other lipids become abnormal in diverse autoimmune diseases. In addition to inducing atherosclerosis, dyslipidemia also accelerates inflammatory response [23]. Therefore, atherosclerosis-associated lipoproteins like HDL-c and LDL are vital risk factors for cardiovascular complications of atherosclerosis in SLE cases. Both HDL-c and Apo-A1 exert a protective effect on atherosclerosis by accelerating cholesterol efflux from macrophages with the assistance of ABC transporters [24, 25]. Apo-B is the main substance of LDL, which promotes atherosclerosis through inducing the phagocytosis of oxidized LDL by macrophages and monocytes. The ratio of Apo-B to Apo-A1 (Apo-B/A1) is usually detected to predict atherosclerosis in clinical practice. A previous study has identified that SLEDAI is negatively correlated to HDL-c, LDL-c, Apo-A1 and Apo-B, but positively correlated to TG and VLDL-C in SLE patients [26]. Consistently, this study showed that SLEDAI was positively correlated to LDL-c, Apo-B and Apo-B/A1, but not to TG, HDL-c, Apo-A1 and IgA. It is suggested that active SLE remarkably accelerates the development of atherosclerosis, and LDL-c, Apo-B and Apo-B/A1, which are closely linked to cardiovascular diseases [27], can be detected for assessing the severity of SLE.

This study for the first time reported the correlation between PGLYRP2 and lipid profile in SLE patients. PGLYRP2 is critical for the innate immunity and chronic inflammation. A high level of PGLYRP2 induces long-term inflammation and RCT damage, thus triggering atherosclerosis and myocardial infarction [28]. Interestingly, this study also showed that PGLYRP2 was negatively correlated to HDL-c and Apo-A1, but positively correlated to Apo-B/A1, indicating that PGLYRP2 serves as an indicator for SLE activity and a predictor for dyslipidemia.

Notably, serum PGLYRP2 level was negatively correlated to IgA level in SLE patients. Serum IgA acts in the activation of multiple antibodies. Through binding to tissue or protein antigens, IgA prevents antigen-induced immune inflammation by eliminating them. How PGLYRP2 influences IgA in SLE patients, however, requires further explorations.

### Study strength and limitations

The increased PGLYRP2 level in SLE patients can be used to predict disease activity and renal damage. SLEDAI is an independent risk factor influencing PGLYRP2 level with a positive correlation. In addition, PGLYRP2 changes with the lipid profile of SLE. It is therefore believed that PGLYRP2 may be used to predict the activity of SLE and the incidence of cardiovascular disease. Several limitations exist in this study. First, a relatively small sample size may restrict the reliability of this study. Second, the potential influences of body weight, comorbidities and current medications on PGLYRP2 level in SLE patients were not assessed. Third, this is a cross-sectional study, and longitudinal studies are necessary to assess the predictive efficiency of PGLYRP2. Finally, great efforts should be made for exploring the mechanism underlying the role of PGLYRP2 in regulating SLE. In the future, multi-center, larger-size prospective studies should be designed to validate the study reliability.

## Conclusions

This study for the first time assessed the correlation between PGLYRP2 and SLE, and provided references for analyzing disease activity of SLE and predicting abnormal lipid metabolism. PGLYRP2 level ascended in SLE patients, and was positively linked to SLEDAI, suggesting that PGLYRP2 may be a biomarker for predicting the activity of SLE. Besides, PGLYRP2 level was correlated to renal damage parameters in SLE patients, suggesting that PGLYRP2 can predict LN damages. SLEDAI was identified positively correlated to LDL-c, Apo-B, and Apo B/A1, and meanwhile, PGLYRP2 was linked to abnormal lipid profile in SLE patients. It is suggested that PGLYRP2 not only served as a biomarker for predicting the severity of SLE, but also the aggravation and risk of abnormal lipid metabolism in SLE patients. Measurement of PGLYRP2 level help guide the early management of SLE and prevention of SLE-associated cardiovascular diseases.

## Data Availability

The datasets used and/or analyzed during the current study are available from the corresponding author on reasonable request.

## Abbreviations

Apo-A1: Apolipoprotein A1
Apo-B: Apolipoprotein B
C3: Complement 3
C4: Complement 4
HDL-C: High-density lipoprotein-cholesterol
LDL-C: Low-density lipoprotein-cholesterol
SLE: Systemic lupus erythematosus
SLEDAI: SLE disease activity index
TG: triglyceride
IgA: Immunoglobulin A
